# Expression of genes associated with BMP signaling pathway in porcine oocytes before and after IVM – a microarray approach

**DOI:** 10.1186/s12958-017-0261-6

**Published:** 2017-06-02

**Authors:** Joanna Budna, Marta Rybska, Sylwia Ciesiółka, Artur Bryja, Sylwia Borys, Wiesława Kranc, Katarzyna Wojtanowicz-Markiewicz, Michal Jeseta, Ewa Sumelka, Dorota Bukowska, Paweł Antosik, Klaus P. Brüssow, Małgorzata Bruska, Michał Nowicki, Maciej Zabel, Bartosz Kempisty

**Affiliations:** 1 Department of Histology and Embryology, Poznan University of Medical Sciences, Swiecickiego 6 St., 60–781 Poznan, Poland; 2 Institute of Veterinary Sciences, Poznan University of Life Sciences, Wolynska 35 St, 60–637 Poznan, Poland; 3 Department of Anatomy, Poznan University of Medical Sciences, Swiecickiego 6 St, 60–781 Poznan, Poland; 4 Department of Obstetrics and Gynecology, University Hospital and Masaryk University, Obilnitrh 11, 602 00 Brno, Czech Republic

**Keywords:** Pig, Oocytes, Microarray, In vitro maturation

## Abstract

**Background:**

The full maturational capability of mammalian oocytes is accompanied by nuclear and cytoplasmic modifications, which are associated with proliferation and differentiation of surrounding cumulus cells. These events are regulated on molecular level by the expression of target genes involved in signal transduction pathways crucial for folliculogenesis and oogenesis. Transforming growth factor beta signaling includes several molecules that are involved in the regulation of oogenesis and embryo growth, including bone morphogenetic protein (BMP). However, the BMP-related gene expression profile in oocytes at different maturational stages requires further investigation.

**Methods:**

Oocytes were isolated from pubertal crossbred Landrace gilts follicles, selected with a use of BCB staining test and analyzed before and after in vitro maturation. Gene expression profiles were examined using an Affymetrix microarray approach and validated by RT-qPCR. Database for Annotation, Visualization, and Integrated Discovery (DAVID) software was used for the extraction of the genes belonging to a BMP-signaling pathway ontology group.

**Results:**

The assay revealed 12,258 different transcripts in porcine oocytes, among which 379 genes were down-regulated and 40 were up-regulated. The DAVID database indicated a “BMP signaling pathway” ontology group, which was significantly regulated in both groups of oocytes. We discovered five up-regulated genes in oocytes before versus after in vitro maturation (IVM): chordin-like 1 (CHRDL1), follistatin (FST), transforming growth factor-beta receptor-type III (TGFβR3), decapentaplegic homolog 4 (SMAD4), and inhibitor of DNA binding 1 (ID1).

**Conclusions:**

Increased expression of CHRDL1, FST, TGFβR3, SMAD4, and ID1 transcripts before IVM suggested a subordinate role of the BMP signaling pathway in porcine oocyte maturational competence. Conversely, it is postulated that these genes are involved in early stages of folliculogenesis and oogenesis regulation in pigs, since in oocytes before IVM increased expression was observed.

## Background

The mammalian cumulus-oocyte complexes (COCs) undergo growth, as well as substantial morphological and biochemical differentiation, during the long stages of folliculogenesis and oogenesis [1]. The biochemical changes include nuclear and cytoplasmic maturation of the oocyte and formation of gap junction connections (GJCs) between the gamete and surrounding somatic cells, which are associated with the bi-directional transport of small substances [2, 3]. The morphological changes involve follicle modifications that occur during their differentiation from primordial and preantral to the antral stage shortly before ovulation. Moreover, during COCs maturation the cumulus cells (CCs), which tightly surround the oocytes, significantly change their structure from compact in immature gametes to expanded, a marker of complete maturation [4]. It is suggested that during in vivo and in vitro mammalian COCs maturation their transcriptomic profile changes. Therefore, some differentially expressed genes may be recognized as new markers of maturational competence [5].

Recently, it was demonstrated that bone morphogenetic proteins (BMPs), apart from its role in bone formation, are involved in porcine folliculogenesis, early embryogenesis and morphogenesis in mammals [6]. Moreover, the BMP family of proteins regulates COCs maturation and achievement of the MII stage [7].

Transforming growth factors beta (TGFβ) are a family of critical proteins that regulate somatic cell proliferation and differentiation both in vivo and in vitro. It has been recognized that the TGF superfamily members regulate important stages of folliculogenesis, oogenesis, and embryogenesis in mammals [8]. Additionally, TGFβ is an upstream activator of SMAD signaling pathway via BMPs kinases complexes [9]. Activation of TGFβ induces subsequent phosphorylation of SMAD2 and SMAD3, finally forming a complex with SMAD4 [10]. The latter, located in the nucleus, regulates expression of inhibitor of DNA binding 1 (ID1) during development and cells differentiation [11, 12]. The ID1 protein contains helix-loop-helix (HLH) architecture and functions in several cell lineages as a regulator of gene transcription following binding to target transcription factors via HLH motif [13]. As the result can regulate growth and differentiation in embryonic tissues [14].

Follistatin (FST) is a negative regulator of follicle growth and function, since it inhibits follicle-stimulating hormone (FSH) release. Interestingly, its increased expression was found in human chondrocytes [15], where chordin-like 1 (CHRDL1) can be also found [16]. Furthermore, both are known as competitive inhibitors of BMPs [17].

Described signaling pathway governs follicle development in the ovary, as well as development and oocyte maturation and competency [18, 19], therefore our aim was to present the influence of BMP signaling pathway on maturation capability of porcine oocytes before and after IVM.

## Methods

### Experimental design

Collected oocytes were exposed to two Brilliant Cresyl Blue (BCB) tests and divided into two groups. The first group (before IVM) included oocytes graded as BCB-positive (BCB^+^) and not subjected to further IVM. The second group (after IVM) included BCB^+^ oocytes which were then in vitro matured, and graded as BCB^+^ after IVM.

### Animals

A total of 45 pubertal crossbred Landrace gilts bred on a commercial local farm were used in this study. They had a mean age of 155 days (range 140–170 days) and a mean weight of 100 kg (95–120 kg). All animals were housed under identical conditions and fed the same forage (depending on age and reproductive status). All experiments were approved by the Local Ethic Committee.

### Collection of porcine ovaries and cumulus-oocyte-complexes (COCs)

The ovaries and reproductive tracts were recovered at slaughter and transported to the laboratory within 40 min. at 38 °C in 0.9% NaCl. To provide optimal conditions for subsequent oocyte maturation and fertilization in vitro, the ovaries of each animal were placed in a 5% fetal bovine serum solution (FBS; Sigma-Aldrich Co., St. Louis, MO, USA) in PBS. Single large follicles (>5 mm) were opened by puncturing with a 5 ml syringe and 20-G needle in a sterile Petri dish, and COCs were recovered. The COCs were washed three times in modified PBS supplemented with 36 μg/ml pyruvate, 50 μg/ml gentamycine, and 0.5 mg/ml BSA (Sigma-Aldrich, St. Louis, MO, USA). COCs were selected under an inverted microscope Zeiss, Axiovert 35 (Lübeck, Germany), counted, and morphologically evaluated using the scale suggested by Jackowska et al. Only COCs of grade I possessing homogeneous ooplasm and uniform, compact cumulus cells were considered for further use, resulting in a total of 300 grade I oocytes (3 x *n* = 50 before IVM group, 3 x *n* = 50 after IVM group).

### *Assessment of oocyte developmental competence by BCB* test

Brilliant Cresyl Blue (BCB) test, which measures activity of glucose-6-phosphate (G6PDH) enzyme, was used for assessment of oocytes’ quality and maturity [20]. The G6PDH enzyme converts BCB stain from blue to colorless. In oocytes that completed the growth activity of the enzyme decreases and the stain cannot be reduced, resulting in blue oocytes (BCB^+^). To perform the BCB staining test, oocytes were washed twice in modified Dulbecco’s Phosphate Buffered Saline (DPBS) commercially supplemented with 0.9 mM calcium, 0.49 mM magnesium, 0.33 mM pyruvate, and 5.5 mM glucose (Sigma-Aldrich, St. Louis, MO, USA), and additionally with 50 IU/ml penicillin, 50 μg/ml streptomycin (Sigma-Aldrich, St. Louis, MO, USA), and 0.4% Bovine Serum Albumin (BSA) [*w*/*v*] (Sigma-Aldrich, St. Louis, MO, USA). They were then treated with 13 μM BCB (Sigma-Aldrich, St. Louis, MO) diluted in DPBS at 38.5 °C, 5% CO_2_ for 90 min. After treatment, the oocytes were transferred to DPBS and washed twice. During washing, the oocytes were examined under an inverted microscope and classified as stained blue (BCB^**+**^) or colorless (BCB^−^). Only the granulosa cell-free BCB^+^ oocytes were used for subsequent molecular analysis (before IVM group) or IVM followed by second BCB test and molecular analysis (after IVM group).

### In vitro maturation of porcine COCs

After the first BCB test, the BCB^+^ COCs were subjected to IVM. Immature oocytes have compact cumulus cell layers that required removal for further oocyte evaluation. Thus, COCs were first incubated with bovine testicular hyaluronidase (Sigma-Aldrich, St. Louis, MO, USA) for 2 min at 38^o^ C to separate cumulus and granulosa cells. Cells were then removed by vortexing the BCB^+^ oocytes in 1% sodium citrate buffer followed by mechanical displacement using a small-diameter glass micropipette (Nichiryo, Nishikata, Japan). The COCs were cultured in Nunclon™Δ 4-well dishes (Thermo Fisher Scientific, Waltham, MA, USA) in 500 μl standard porcine IVM culture medium: TCM-199 (tissue culture medium) with Earle’s salts and *L*-glutamine (Gibco BRL Life Technologies, Grand Island, NY, USA), supplemented with 2.2 mg/ml sodium bicarbonate (Nacalai Tesque, Inc., Kyoto, Japan), 0.1 mg/ml sodium pyruvate (Sigma-Aldrich, St. Louis, MO, USA), 10 mg/ml BSA (Bovine Serum Albumin) (Sigma-Aldrich, St. Louis, MO, USA), 0.1 mg/ml cysteine (Sigma-Aldrich, St. Louis, MO, USA), 10% (*v*/v) filtered porcine follicular fluid, and gonadotropin supplements at final concentrations of 2.5 IU/ml hCG (human Chorionic Gonadotropin) (Ayerst Laboratories, Inc., Philadelphia, PA, USA) and 2.5 IU/ml eCG (equine Chorionic Gonadotropin) (Intervet, Whitby, ON, Canada). Wells were covered with a mineral oil overlay and cultured for 44 h at 38 °C under 5% CO_2_. After cultivation, the BCB staining test was performed, and BCB^+^ oocytes were used for further experiments.

### RNA extraction from porcine oocytes

Total RNA was extracted from samples using TRI Reagent (Sigma, St Louis, MO, USA) and RNeasy MinElute cleanup Kit (Qiagen, Hilden, Germany). The amount of total mRNA was determined from the optical density at 260 nm, and the RNA purity was estimated using the 260/280 nm absorption ratio (higher than 1.8) (Nano Drop spectrophotometer, Thermo Scientific, ALAB, Poland). The RNA integrity and quality were checked on a Bioanalyzer 2100 (Agilent Technologies, Inc., Santa Clara, CA, USA). The resulting RNA integrity numbers (RINs) were between 8.5 and 10 with an average of 9.2 (Agilent Technologies, Inc., Santa Clara, CA, USA). The RNA in each sample was diluted to a concentration of 100 ng/μl with an OD260/OD280 ratio of 1.8/2.0. From each RNA sample, 500 ng of RNA was taken. The remaining amount of isolated RNA was used for the RT-qPCR study.

### Microarray expression analysis and statistics

The Affymetrix procedure was previously described by Trejter et al. [21]. Total RNA (100 ng) from each pooled sample was subjected to two rounds of sense cDNA amplification (Ambion® WT Expression Kit). The cDNA was used for biotin labeling and fragmentation by Affymetrix Gene Chip® WT Terminal Labeling and Hybridization (Affymetrix). Biotin-labeled fragments of cDNA (5.5 μg) were hybridized to Affymetrix® Porcine Gene 1.1 ST Array Strip (48 °C/20 h), where the expression profile of 12,258 porcine transcripts was examined. Microarrays were then washed and stained according to the technical protocol using the Affymetrix Gene Atlas Fuidics Station. The array strips were scanned employing Imaging Station of the Gene Atlas System. Preliminary analysis of the scanned chips was performed using Affymetrix Gene Atlas TM Operating Software. The quality of the gene expression data was checked according to quality control criteria provided by the software. Obtained CEL files were imported into downstream data analysis software.

All analyses were performed using Bioconductor software based on the statistical R programming language. For background correction, normalization, and summation of raw data, the Robust Multiarray Averaging (RMA) algorithm implemented in “affy” package of Bioconductor was applied. Biological annotation was taken from Bioconductor “oligo” package where the annotated data frame object was merged with a normalized data set, leading to a complete gene data table. Statistical significance of the analyzed genes was performed by moderated t-statistics from the empirical Bayes method. Obtained *p*-values were corrected for multiple comparisons using the Benjamini and Hochberg’s false discovery rate. Selection of significantly changed gene expression values (differentially expressed genes) was based on a *p*-value below 0.05 and an expression fold higher than |2|. The homogeneity of analyzed groups was checked by a principal component analysis (PCA) algorithm incorporated in “rgl” Bioconductor package.

Differentially expressed genes were subjected to the selection of genes associated with BMP signaling pathways. Differentially expressed gene lists (separated for up- and down-regulated) were uploaded to DAVID software (Database for Annotation, Visualization and Integrated Discovery) where differentially expressed genes belonging to “BMP signaling pathway” gene ontology biological process group (GO BP) were obtained [22]. Expression data of these genes were subjected to a hierarchical clusterization procedure and presented as a heatmap.

Interactions between differentially expressed genes/proteins belonging to “BMP signaling pathway” ontology group were investigated by STRING10 software (Search Tool for the Retrieval of Interacting Genes) [23]. List of gene names was used as query for interaction prediction. Search criteria were based on co-occurrences of genes/proteins in scientific texts (text mining), co-expression, and experimentally observed interactions. The results of this analysis generated a gene/protein interaction network where the intensity of the edges reflects the strength of the interaction score. In addition to interaction prediction, STRING also performs functional enrichments of GO terms based on previously uploaded gene sets from “BMP signaling pathway” GO BP term.

### Real-time quantitative polymerase chain reaction (RT-qPCR) analysis

RT-qPCR analysis was performed in order to validate microarray results, using both the same RNA samples used for PCR and microarray profiling experiments.

Total RNA was isolated from oocytes before or after IVM. The RNA samples were re-suspended in 20 μl of RNase-free water and stored in liquid nitrogen. RNA samples were treated with DNase I and reverse-transcribed (RT) into cDNA. RT-qPCR was conducted in a Light Cycler real-time PCR detection system (Roche Diagnostics GmbH, Mannheim, Germany) using SYBR® Green I as a detection dye, and target cDNA was quantified using the relative quantification method. The relative abundance of CHRDL1, FST, TGFβR3, SMAD4, and ID1transcripts in each sample was standardized to the internal standards. For amplification, 2 μl of cDNA solution was added to 18 μl of QuantiTect® SYBR® Green PCR (Master Mix Qiagen GmbH, Hilden, Germany) and primers (Table 1). One RNA sample of each preparation was processed without the RT-reaction to provide a negative control for subsequent PCR.

**Table 1:** Oligonucleotide sequences of primers used for RT-qPCR analysis

Transcript	Sequence (5′-3′ direction)	Gene accession no.	Product size (bp)	Efficiency
CHRDL1	AACAATGCCTGTGTATGAGT TCTGGGCTTCTCCTTCAGT	XM_005673817.2	242	91%
FST	GAGCCCACCTCCTCAGGAC TCTCAGGGCACAGCTCATCG	NM_001003662.1	238	94%
TGFβR3	TGATCCACCATGAAGTGCAGT TGCCTTCCTGCGCTGTCTC	NM_214272.1	190	108%
ID1	AGCTGAACTCGGAATCCCAA TTCAGCGACACAAGATGCGAT	NM_001244700.1	147	107%
SMAD4	CCAAGTGCATATATAAAGGTCT AGCCTTTCACAAAACTCATCC	XM_013985326	235	98%
PBGD	GAGAGTGCCCCTATGATGCT ATGATGGCACTGAACTCCT	NM_001097412.1	214 bp	97%
B-ACTIN	GGGAGATCGTGCGGGACAT CGTTGCCGATGGTGATGAC	DQ845171	141 bp	99%
18S rRNA	GTGAAACTGCGAATGGCTC CCGTCGGCATGTATTAGCT	AB117609	105 bp	97%

## Results

From whole sets of 12,258 analyzed transcripts, 379 genes were down-regulated whereas 40 were up-regulated in relation to the oocyte transcriptome before and after the in vitro maturation procedure. Principal Component Analysis (PCA) confirmed the homogeneity of the groups, since we obtained two separated clusters corresponding to tested experimental groups (before and after IVM).

**Fig. 1:**
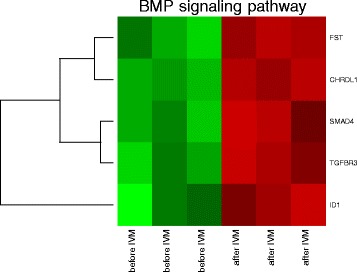
Heat map representing differentially expressed genes belonging to the “BMP signaling pathway” - functional category from DAVID GEOTERM BP database. Arbitrary signal intensity acquired from microarray analysis is represented by colors (green – higher, red - lower expression). Log2 signal intensity values for any single gene were resized to Row Z-Score scale (from −2 - the lowest expression to +2 - the highest expression for single gene)

DAVID software extracted five genes sets belonging to “BMP signaling pathway” gene ontology biological process term (GO BP). The set of genes consisting of CHRDL1, FST, TGFβR3, SMAD4, and ID1 was subjected to hierarchical clusterization procedure and presented as a heatmap. Arbitrary signal intensity acquired from microarray analysis was represented by green (higher expression) and red (lower expression) colors. Log2 signal intensity values for any single gene were resized to Row Z-Score scale (from −2 - the lowest expression to +2 - the highest expression for single gene) (Fig. 1). The set of differentially expressed genes belonging to “BMP signaling pathway” GO BP term category is presented in Table 2 where their symbols, names, fold changes, and corrected *p*-values are shown.

**Table 2:** Fold changes and adjusted *p*-values of differentially expressed genes

Gene symbol	Gene name	Fold change	Adj. *p* value
CHRDL1	chordin-like 1	−7,18	0,00005
TGFβR3	transforming growth factor, beta receptor III	−5,09	0,00041
FST	follistatin	−4,45	000036
ID1	Inhibitor of DN binding 1, dominant negative helix-loop-helix protein	−2,98	0,00397
SMAD4	SMAD family member 4	−2,72	0,00124

Fold changes and adjusted *p*-values of differentially expressed genes belonging to the “BMP signaling pathway” functional category from DAVID GEOTERM BP database. Symbols and names of the selected genes are also shown

Our analyses of differentially expressed genes, belonging to the BMP signaling pathway GO, with a use of STRING database revealed only weak interactions between ID1, SMAD4, and FST genes. We applied prediction methods such as text mining, co-expression and experimentally observed interactions, and found only weak interaction of protein homology. Strength of the interaction was reflected by the intensity of the edges.

STRING-generated functional enrichment of GO terms showed the top five GO terms that also belong to “BMP signaling pathway” GO BP. These terms include those that are very similar to “BMP signaling pathway” such as: “response to BMP”, “cellular response to BMP stimulus”, “transmembrane receptor protein serine/threonine kinase signaling pathway”, and “regulation of transmembrane receptor protein serine/threonine kinase signaling pathway”, with their GO ID (pathway ID), GO term description (pathway description), and number of the genes belonging to appropriate category (count in gene set) (Table 3).

**Table 3:** Top five GO categories formed by differentially expressed genes

Biological Process (GO)		
Pathway ID	Pathway Description	Count in gene set
GO: 0030509	BMP signaling pathway	4
GO: 0071772	Response to BMP	4
GO: 0071773	Cellular response to BMP stimulus	4
GO: 0007178	Transmembrane receptor protein serine/threonine kinase signaling pathway	4
GO: 0090092	Regulation of transmembrane receptor protein serine/threonine kinase signaling pathway	4

Top five GO categories formed by differentially expressed genes belonging to the “BMP signaling pathway” ontology group. GO categories were generated in STRING software. GO ID (pathway ID), GO term description (pathway description), number of the genes belonging to appropriate category (count in gene set) are shown

**Fig. 2:**
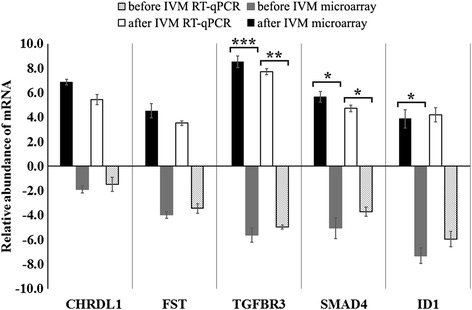
Validation of microarray data by RT-qPCR. Comparison of gene expression analysis of oocytes before IVM and after IVM using microarray assay and RT-qPCR. RT-qPCR analysis was normalized to the expression of three housekeeping genes (PBGD, β-actin, 18S rRNA). Error bars represent the standard error of the mean (SEM) for groups of oocytes. Statistically significant differences are presented as: *p < 0.05, **p < 0.01, and ***p < 0.001

The result from the RT-qPCR revealed increased expression of CHRDL1, FST, TGFβR3, SMAD4, and ID1 in porcine oocytes before IVM as compared to analysis after IVM. The RT-qPCR assay confirmed the fold change and significance of microarray expression profiling. Figure 2 shows comparison of both techniques with their respective fold changes for each gene.

## Discussion

Maturation capability is defined as the ability of female gametes to undergo nuclear and cytoplasmic maturation [24]. This occurs when oocytes achieve the MII stage and RNA accumulates, creating the template for further protein synthesis during early embryogenesis [25]. It has been clearly demonstrated in several species of mammals, including domestic pigs, that only after proper maturation, the oocytes are fully fertilizable [26]. It has been also suggested that the maturation period is crucial for normal zygote formation and embryonic growth in the preimplantation stage [27]. Although in vitro culture (IVC) systems are applied to mimic in vivo conditions, the proportion of fully mature and fertilizable oocytes after IVC in numerous mammalian species is still unsatisfactory [28]. Recently, several studies aimed to find new molecular markers of oocyte maturation ability in order to improve IVC models and increase the number of fully mature gametes [29–33]. Therefore, in this study we performed gene expression analysis in porcine oocytes before and after IVM in order to define new molecular markers of female gamete maturation capability. Using microarray analysis, we selected genes related to the BMP family that were significantly up-regulated before IVM as compared to after IVM.

Chordin-like 1 (CHRDL1), also known as ventroptin (VOPT), is a protein involved in retina and tectum development, is crucial for topographic retinotectal projection, and is a known BMP4 antagonist [34]. Recent findings by Webb et al. [35] showed the expression of ventroptin in the developing human cornea and neural retina as early as week 7 of gestation. Additionally, they observed ventroptin expression in fetal organs such as the cerebellum as well as the prefrontal and occipital neocortex. These data suggest that CHRDL1 may belong to proteins involved in morphogenesis and organogenesis in mammals. The results from our experiments indicated CHRDL1 was significantly up-regulated before IVM as compared to oocytes analyzed after IVM. Since CHRDL1 is a secreted protein expressed mostly in mesenchymal tissue during morphogenesis and organogenesis [36], its expression may be possibly related to embryos as opposed to mature oocytes. Moreover, higher expression of CHRDL1 in porcine oocytes before IVM let us assume that BMP-related morphogenesis may be significantly associated with early folliculogenesis in immature oocytes. Bachiller et al. [37], found that *CHRDL1* (−/−) mice died during embryogenesis or perinatelly, what let us assume that this morphogenesis–related gene expression is more likely associated with embryo growth and development than with achievement of maturation capability in porcine oocytes.

A significant role of the TGF signaling pathway during early morphogenesis and organogenesis has also been determined [38]. In this study, we observed that induction of the BMP signaling pathway can be also associated with up-regulated gene expression of TGF family member follistatin (FST) and genes related to TGF signaling, such as transforming growth factor-beta receptor-type III (TGFβR3). Additionally, altered expression of the mother against decapentaplegic homolog 4 (SMAD4) transcript, known as the main mediator of TGF-beta (TGFβ) and BMP1 signal transduction, was also observed.

Recently, Inman et al. [39] reported that TGFβ receptor (TGFβR) activity is required for nuclear SMAD activation, which regulates induction of TGFβR transcription. This bi-directional transport of SMADs/TGFβR between the nucleus and cytoplasm provides the information regarding signaling pathways and events leading to the transcriptional activation of target genes. It has been suggested that activing- and activing receptor-related systems are involved in regulatory processes responsible for the maturational capability of oocytes [40].

The results of our microarray experiments clearly demonstrated up-regulation of all three members of TGF family: FST, TGFβR3, and SMAD4 in porcine oocytes before IVM compared to those analyzed after IVM. We therefore hypothesize that FST, TGFβR3, and SMAD4 could be involved in oocyte maturational competence, as well as induction of the TGF/TGFR signaling pathway. The latter could significantly improve the oocyte-follicular cell bi-directional shuttling. Our results may indicate that expression and likely release of FST out of the oocyte improve follicular cell growth and differentiation. Similar to Wang and Ge [41], we observed TGFβ–related genes are up-regulated in oocytes before compared to after IVM gametes. Al-Edani et al., also observed up-regulated expression of TGFβR3 gene in human cumulus cells, supposedly as the result of enhanced angiogenesis, playing essential role in late stages of folliculogenesis [42]. Furthermore, Rodriques et al., found TGFβ, TGFβR I and R II mRNA in oocytes of all follicle stages as well as in granulosa cells of primary and secondary follicles in caprine [43]. That is why, we cannot exclude the existence of a TGF/TGFβ/TGFβR signaling cascade between oocytes and follicular cells, which maintains significantly higher activity at early stages of oogenesis and folliculogenesis.

It is likely that activation of the TGF signaling pathway in both oocytes and follicular cells is necessary for proper growth, development, and maintenance of proper maturational capability of porcine oocytes [44]. Results obtained by Guéripel et al. can confirm this hypothesis, since they observed increased expression of TGFβ signaling pathway genes and proteins in mice exposed to FSH an LH treatment. Significant expression of TGFβR I and R II in theca interna, whereas much lower expression in GCs was found. The Smads exhibited strong expression in oocytes, GCs, and luteal cells but lower expression in the theca interna. Among the Smads, Smad4 had the highest expression [45].

It was well defined that oocyte maturational competence is significantly regulated by bi-directional shuttling of oocytes and somatic cumulus cells [46]. The transport of molecules between these cells improves the metabolic status and significantly regulates the maturation ability of oocytes. Moreover, our recent studies clearly demonstrated that oocyte-cumulus cross-talk supports somatic cell proliferation and differentiation in vitro [47]. Although the important role of CCs surrounding oocytes in gametes maturational competence achievement is widely known [48], there is relatively little data indicating the molecular basis of this process. Indeed, Salhab et al. used microarray assays to identify a transcriptomic profile in bovine CCs. Among 472 differentially expressed transcripts in CCs, TGFβ signaling GO was up-regulated, whereas MAP kinases pathway GO was down-regulated. Additionally, the protein assays showed an increased abundance of Smad4 in CCs after oocyte’s IVM. The phosphorylation status of SMAD2, MAPK3/1, and MAPK14, but not MAPK8, was higher in the cells after IVM as compared to immature complexes. They concluded that in vitro maturation leads to increased activity of the TGFβ and MAPK signaling pathways, simultaneous with decreased oocyte quality [49].

Contrary to these results, the current study observes significantly lower expression of TGF/TGFβ signaling related genes. Thus, we suspect an important role of this ontological group in the early stages of folliculogenesis and oogenesis, likely leading to maturational competence of oocytes in pigs. Although, the function of Smad4 as the mediator of early embryogenesis is not entirely known, the role of SMAD4 as a regulator of SMAD2/3- and SMAD1/5/8 signaling pathways activation and FST expression was recently documented in cattle oocytes by Lee et al. Contrary to our observations, they found that Smad4 mRNA was significantly higher during oocyte’s IVM with a maximum at 2-cell stage embryos and 8-cell stage to the lower level at blastocyst, concluding SMAD4 may be recognized as the main factor required for normal embryogenesis in cattle at 8-cell, 16-cell, and blastocyst stage [50]. Taking into account also our observations, it is possible that SMAD4 is an important component of the TGFβ signaling pathway responsible for regulation of proper oogenesis and embryogenesis as well as achievement and maintenance of normal maturational status in both pigs and cattle.

It was well recognized in several species of mammals that the proper achievement of oocyte maturational capability is significantly orchestrated by cumulus cell proliferation and differentiation both in vivo and in vitro. Hogg et al. [51], examined ovine ovary, and found ID 1–4 expression in granulosa and theca cells in ovarian follicles during their development. Finally, they hypothesized that ID proteins may play a key role in steroidogenic cells’ regulation of growth and differentiation. This observation together with ID1 known functions of proliferation maintenance and differentiation inhibition [14], may indicate that ID1 can be involved in transcriptional regulation essential for normal folliculogenesis. Although the role of ID1 as an important transcriptional activating factor in the mediation of cellular growth, development, proliferation, and differentiation is well known [14], there exists only one description regarding an ID1 expression profile and oocyte maturation. Similarly to us, Blaha et al. using microarray assay investigated the effect of FSH administration on gene expression patterns in porcine cumulus cells. They found that FSH administration led to increased expression of genes encoding transcription factors including ID1. Finally, they concluded that FSH-induced expression of genes is responsible for regulation of cumulus cell differentiation and the events leading to successful oocytes in vitro maturation [52].

## Conclusions

Higher expression of CHRDL1, FST, TGFβR3, SMAD4, and ID1 genes before IVM, as compared to oocytes analyzed after IVM, points these genes may be potential mediators of fully maturational competent gamete formation both in the nucleus and cytoplasm. We hypothesize that BMP signaling pathway genes influence regulatory processes at early stages of porcine oogenesis, however significance of this finding needs further investigation with a use of protein assays.

## Abbreviations

ALK: Activing-related kinases
BMP: Bone morphogenetic protein
CCs: Cumulus cells
CHRDL1: Chordin-like 1
COCs: Cumulus-oocyte complexes
DAVID: Database for annotation, visualization and integrated discovery
FST: Follistatin
GCs: Granulosa cells
GJCs: Gap junction connections
GO BP: Gene ontology biological process group
HLH: Helix-loop-helix (architecture)
ID1: Inhibitor of DNA binding 1
IVM: In vitro maturation
PCA: Principal component analysis
RMA: Robust Multiarray averaging
SMAD: Decapentaplegic homolog
STRING: Search tool for the retrieval of interacting genes
TGFβ: Transforming Growth Factor beta
TGFβR: Transforming Growth Factor Beta Receptor
VOPT: Ventroptin
